# The function and evolutionary significance of a triplicated Na,K-ATPase gene in a toxin-specialized insect

**DOI:** 10.1186/s12862-017-1097-6

**Published:** 2017-12-15

**Authors:** Jennifer N. Lohr, Fee Meinzer, Safaa Dalla, Renja Romey-Glüsing, Susanne Dobler

**Affiliations:** 1 Universität Hamburg, Biozentrum Grindel, Zoologisches Institut, Martin-Luther-King Pl. 3, 20146 Hamburg, Germany; 2 Department of Genetics, Evolution and Environment, Institute of Healthy Ageing, University College London, WC1E 6BT, London, UK

**Keywords:** Milkweed bug, Coevolution, Cardenolides, RNAi, Gene expression, Gene duplication, *Oncopeltus fasciatus*

## Abstract

**Background:**

The Na,K-ATPase is a vital animal cell-membrane protein that maintains the cell’s resting potential, among other functions. Cardenolides, a group of potent plant toxins, bind to and inhibit this pump. The gene encoding the α-subunit of the pump has undergone duplication events in some insect species known to feed on plants containing cardenolides. Here we test the function of these duplicated gene copies in the cardenolide-adapted milkweed bug, *Oncopeltus fasciatus,* which has three known copies of the gene: α1A, α1B and α1C.

**Results:**

Using RT-qPCR analyses we demonstrate that the α1C is highly expressed in neural tissue, where the pump is generally thought to be most important for neuron excitability. With the use of in vivo RNAi in adult bugs we found that α1C knockdowns suffered high mortality, where as α1A and α1B did not, supporting that α1C is most important for effective ion pumping. Next we show a role for α1A and α1B in the handling of cardenolides: expression results find that both copies are primarily expressed in the Malpighian tubules, the primary insect organ responsible for excretion, and when we injected either α1A or α1B knockdowns with cardenolides this proved fatal (whereas not in controls).

**Conclusions:**

These results show that the Na,K-ATPα gene-copies have taken on diverse functions. Having multiple copies of this gene appears to have allowed the newly arisen duplicates to specialize on resistance to cardenolides, whereas the ancestral copy of the pump remains comparatively sensitive, but acts as a more efficient ion carrier. Interestingly both the α1A and α1B were required for cardenolide handling, suggesting that these two copies have separate and vital functions. Gene duplications of the Na,K-ATPase thus represent an excellent example of subfunctionalization in response to a new environmental challenge.

**Electronic supplementary material:**

The online version of this article (10.1186/s12862-017-1097-6) contains supplementary material, which is available to authorized users.

## Background

The Na,K-ATPase is a critical animal cell protein, responsible for generating the ion gradients needed to maintain resting potentials and the excitability of neurons, as well as regulating overall cell volume. The pump has numerous additional functions, such as tight junction formation and cell signaling [1–3]. It is an active transporter, which uses ATP to pump three sodium ions out of the cell for each two potassium ions into the cell [4]. The protein consists of up to three subunits: alpha (α), beta (β), and gamma (γ). The α-subunit, on which we focus in this paper, performs the actual pumping function, whereas the β-subunit is important in modulating the activity of the pump [4]. Little is known about the γ-subunit, which has so far only been found in vertebrates, but may modulate the kinetic characteristics of the pump [5–7].

The activity of the Na,K-ATPase can be regulated by a family of inhibitors known as cardiac glycosides, which bind to and inhibit the pumping function of the Na,K-ATPase and are toxic at high doses [4, 8]. Due to their ability to act on the contractile force of cardiac muscle, such cardiac glycoside containing plants have been used to treat heart failure dating back to the 1700s [9]. Cardiac glycosides are toxins produced by plant and animal species to ward off predators [10, 11]. For example, milkweed, foxglove, and oleander plants produce cardiac glycosides (of the cardenolide type) that protect against herbivorous insects and bufonid toads secrete cardiac glycosides (of the bufadienolide type) from their parotoid glands for similar purposes against vertebrate predators [10, 12]. Interestingly, recent research in mammals has revealed the presence of endogenous cardiac glycosides of both types, which are synthesized in the adrenal gland [9], and suggests that these defense compounds are mimics of endogenous hormones.

Despite their toxicity several insect species, like the large milkweed bug, *Oncopeltus fasciatus* investigated here, sequester cardenolides in their own body and thus profit from their deterrent and toxic properties [10, 11]. Plants producing these toxins contain a wide battery of cardenolides with differences in polarity and correspondingly in toxicity. Yet, the types and the specific identity of the cardenolides sequestered remain unknown. Indeed, only a small number of the naturally occurring cardenolides found in plant and insect taxa have been identified.

**Fig. 1:**
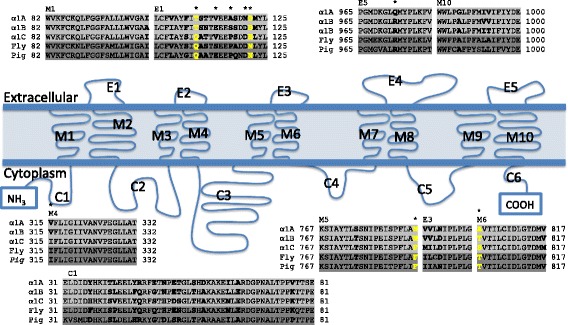
Schematic representation of the 3 Na,K-ATPase α-subunits with partial regions of the alignment of important loops and segments (M_1_-M_10_ are the transmembrane domains, E_1_-E_5_ the extracellular domains and C_1_-C_6_ the intracellular domains of the protein). Stars above specific residues indicate a site thought to be involved in cardenolide resistance and yellow sites are those confirmed to have importance via assays in cell culture. α1A-C are the three gene copies from *O. fasciatus*, fly = *Drosophila melanogaster*, pig = *Sus scrofa*. The pig sequence is included as it was used to determine the structure of this protein

Across several insect lineages, gene duplications of the Na,K-ATPase α-subunit have generated multiple copies of the gene [13, 14]. Here we focus on the large milkweed bug (*O. fasciatus*), which has three known copies of the Na,K-ATPase α1-subunit gene [13]; hereafter referred to as the α1A, α1B and α1C copies. Previous work on these three copies has identified several amino acid substitutions in the region that codes for the cardenolide binding pocket of the Na,K-ATPase [13–15]. Specifically, all three copies have a substitution at position 122, whereas α1A and α1B have two additional substitutions at position 111 and 786, and finally α1A and α1C have further, but different substitutions at position 797 (Fig. 1; [14]). When these substitutions were introduced into the *Drosophila* Na/K-ATPase via mutagenesis for expression in cell culture and followed by a cardenolide-sensitivity assay, it was shown that the substitutions found in α1A and α1B result in a pump more resistant to cardenolides than those found in α1C [14]. Yet, the same cardenolide resistance conferring substitutions found in the α1A and α1B also reduce the pump’s overall activity, thus suggesting that the resistant α1A and α1B can continue to pump at high cardenolide concentrations, but only very slowly. This is in contrast to substitutions found in the α1C, which result in a pump inhibited at high cardenolide concentrations, but which retains an activity comparable to that of sensitive pumps found in other species [14].

Here, we investigate if these differences between gene copies, as suggested by in vitro studies in the *Drosophila* pump, translate into biological differences in vivo in *O. fasciatus*. We use RNAi coupled with a gene-expression study via RT-qPCR to test the function of the original Na,K-ATPase α-subunit gene copies of *O. fasciatus*. We look specifically at the survival of copy-specific knockdowns, as well as their ability to tolerate cardenolides. We also look at the spectrum and amount of cardenolides found in milkweed bugs and go on to look for differences in the expression of the Na,K-ATPase when bugs are challenged with cardenolides. Our results lend exciting insights into how the genotypes of these duplicated gene copies determine their phenotype and what specific subfunctionalizations have evolved.

## Methods

### Sequences comparisons

To compare the nucleotide and amino acid diversity of the 3 Na,K-ATPase copies from *O. fasciatus* (α1A: JQ771520, α1B: JQ771519, α1C: JQ771518), we performed an alignment in Mega (v6.06). We also included the Na,K-ATPase sequence for the fruit fly *Drosophila melanogaster* (AF044974) and the pig *Sus scrofa* (NM_214249) as comparative taxa, known to be sensitive to cardenolides. Thirty amino acids were removed from the 5’end of the alignment as not all of the copies were complete in this area. The full alignment can be found in the Additional file 1: sequence alignment. The alignment was then analysed for pairwise differences at both the nucleotide and amino acid level (Table 1).

**Table 1:** Pairwise differences for the three *O. fasciatus* Na/K-ATPase-α1 gene copies

	Amino acid / Nucleotide pairwise differences				
	*O. fasciatus* α1A	*O. fasciatus* α1B	*O. fasciatus* α1C	*D. melanogaster*	*S. scrofa*
*O. fasciatus* α1A	X	0.16	0.22	0.29	0.34
*O. fasciatus* α1B	0.08	X	0.19	0.28	0.34
*O. fasciatus* α1C	0.12	0.08	X	0.26	0.34
*D. melanogaster*	0.15	0.12	0.10	X	0.28
*S. scrofa*	0.25	0.24	0.24	0.23	X

Values below the central X-line are pairwise differences at the amino acid level and values above the central X-line are pairwise differences at the nucleotide level

### Origin and handling of milkweed bugs

*Oncopeltus fasciatus* bugs were collected from the surrounding of the Cornell campus in Ithaca, New York in 2010. Adult bugs were transported to our lab at the University of Hamburg, Germany shortly thereafter. Two sets of cultures were then established: one fed exclusively on sunflower seeds and the other fed on a mixture of milkweed seeds (*Asclepias syriaca;* two different populations*)* and sunflower seeds. In 2014 a second set of adult bugs collected from a field near the University of Chicago, Illinois was added into the two cultures to increase the genetic diversity of the laboratory culture and to break up extended generations of inbreeding.

For standardized use in RNAi experimentation and to minimize fluctuations in gene expression, all bugs used in the following experiments were handled as follows: L5 stage larvae were separated from mass cultures as single individuals into 100 ml plastic vials. Once the L5 larvae molted to the adult stage we waited for 5 days before injecting the bugs with dsRNA probes.

### Comparisons of gene expression between treatments

Three separate relative RT-qPCR analyses were run: (1) differences in expression of the α1A, α1B and α1C copies across the milkweed bugs tissue types (muscle, Malphigian tubules, gut, nervous tissue and ovaries); (2) differences in expression between the RNAi treatments (negative control – injected with buffer, positive control – injected with knockdown for *eGFP*, knockdown for α1A, knockdown for α1B, knockdown for α1C); (3) differences in expression between control bugs and those challenged with cardenolides either as a food source (sunflower diet as control, *A. syriaca* as treatment) or as a haemolymph injection (0.9% NaCl injection as control, ouabain injection as treatment).

### RNA extraction and quantitative RT-PCR

Once the bugs from the various treatments were ready for preparation and RNA extraction they were first frozen at −80 **°**C. Those bugs for which the whole-body-expression level was being assessed were then placed on dry ice and ground up using a pestle (see following paragraph for details). For those bugs where the individual tissue types were being investigated, they were placed in saline solution in a glass preparation stage surrounded by ice. The preparation stage was then placed under the dissecting microscope, where the tissues were carefully teased apart using two sets of dissecting pincettes. The body of the bug was first positioned dorsally so as to remove the wings. Then, ventrally the abdomen was carefully split open along one side and exoskeleton removed to reveal the digestive organs. At this point the fat body tissue, the Malphigian tubules and the gut could be isolated into individual Eppendorf tubes on ice. To retrieve the nervous and muscle tissue the midsection and head of the bug was pulled open, also ventrally. Due to the small amount of tissue yielded per bug, pooled samples from three bugs were used. Each tissue was then homogenized individually, as described below.

RNA was extracted using the RNeasy kit (Qiagen, Hilden, Germany) with a genomic DNA eliminator column, according to kit instructions. Whole bugs were homogenized in RTL buffer using a plastic pestle, whereas isolated tissues were homogenized using an ultrasound tissue homogenizer (OMNI Sonic Ruptor 400) for 15 min at maximum resonance. Isolated RNA was resuspended in 50 μl nuclease free water (30 μl for tissue samples) and quality was checked using an RNA gel and quality spectrum ratios (Nanodrop 2000, Thermo Fisher Scientific). We synthesized cDNA using Superscript III (Invitrogen) according to kit instructions to a final concentration of 100 ng/μl. The cDNA quality check was performed using TAE-agarose-electrophoresis (1% gel).

Quantitative RT-PCR was performed using SYBR Green qPCR Master Mix (Thermo Fisher Scientific) according to the manufacturer’s instructions and a StepOne Real-Time PCR system (Thermo Fisher Scientific). A total of 20 ng RNA was run per well for a final concentration of 1 ng/μl. Primers for the reference genes were constructed using the Primer3 online toolkit [16], and efficiencies were calculated using the standard dilution-series method [17]. We used existing α1A, α1B and α1C qPCR primers of the *O. fasciatus* Na,K-ATPase α-subunit [13]. Analyses were performed according to the MIQE-guidelines [17]. The details of the RT-qPCR primers can be found in Additional file 2: Table S1.

### Reference genes for use in RT-qPCR normalization

We designed and tested seven possible reference genes to use in the normalization of our RT-qPCR data sets (Additional file 2: Table S1). We used NormFinder v3.4 [18] to investigate the stability (M) of raw non-normalized RT-qPCR data across the five tissue treatments (muscle, nerve, Malphigian tubules, gut and ovaries; Additional file 3: Figure S1a), five RNAi treatments (negative control – not injected, positive control – eGFP injected, knockdown α1A, knockdown α1B, knockdown α1C; Additional file 3: Figure S1b), and four cardenolide challenge treatments (bugs fed on sunflower seeds, bugs fed on milkweed seeds, bugs injected with a buffer solution, bugs injected with the cardenolide ouabain; Additional file 3: Figure S1c). Using NormFinder we calculated the expression stability of each gene using the logarithm of mean q = eff^ct-ctmin^ values and then ranked the reference genes from least stable to most stable (Additional file 3: Figure S1). Actin was the most stable gene over all the treatment analyses and was thus used as our gene for normalization. Primers used for RT-qPCR for the α1A, α1B and α1C were designed in a previous study [13] and they performed similar to the reference gene designed here in terms of efficiency and amplification (see excel files in Additional file 4: for details and calculations).

### RNAi design, implementation and confirmation

A 540 bp region near the 3′ end of the Na,K-ATPase α-subunit was used to create dsRNA probes for RNAi (for primer information see Additional file 2: Table S2). As a positive control, a 433 bp segment of the e*GFP* sequence was amplified from pcDNA3.1/CT-GFP-TOPO (Invitrogen). We used transcriptome and genome searches and sequence comparisons to ensure that no off-target effects were predicted for other genes of *O. fasciatus*. Monitoring of the reference genes via quantitative RT-PCR was used as an additional control for off-target effects and for data normalization.

Sequence-confirmed PCR products of the amplified sequences were subject to an in vitro-transcription assay according to instructions from the Ambion MEGAscript RNAi kit (Life Technologies, Darmstadt, Germany; see Table 1 for primer sequences). The resulting dsRNA was eluted after nuclease digestion two times with 50 μl of injection buffer (3.5 mM Tris-HCl, 1 mM NaCl, 50 nM Na_2_HPO_4_, 20 nM KH_2_PO_4_, 3 mM KCl, 0.3 mM EDTA, pH 7.0). The quality of the dsRNA was checked by TBE-agarose-electrophoresis (1% gel).

Age-standardized adult bugs (5 days post adult morph) were injected with 2 μg of dsRNA (total volume 2 μl) using a Hamilton injector. Injections were made directly into the haemolymph between the two posterior most abdominal segments. For all experiments we included a positive control, which was injected with a *gfp* dsRNA sequence and a negative control, which was injected with buffer only. Expression levels were monitored using RT-qPCR as described above. There was large variation in knockdown success between replicates, probably due to the similarity of the three gene copies and the long length of the dsRNA segment used for RNAi. Thus, each injected individual had to be verified as successful or not via RT-qPCR using extractions from legs or freshly dead animals. Only knockdowns where the normalized expression level (normalized Ct) value was reduced by 90% or more as compared to the positive control were included in our analyses.

### Cardenolide assays for survivorship

Solutions of two cardenolides, ouabain and digoxin, were injected into the haemolymph of adult bugs using a Hamilton injector, as for the dsRNA described above. Stock solutions were prepared at a concentration of 5 mg/ml with 1% NaCl and 12% ethanol (the latter to increase dissolvability). A total volume of 2 μl was injected for final cardenolide content of 10 μg. A mock solution of 1% NaCl and 12% ethanol was used for the positive control treatment. Negative controls were not injected with any solution. Survivorship was recorded 48-h post injection.

To test for differences in the number of individuals that survived injection with both dsRNA for RNAi silencing, as well as injection with the cardenolides ouabain and digoxin, we used ANOVA tests in R [19]. Post-hoc Tukey HSD tests were used to evaluate pairwise differences within the model.

### High-performance liquid chromatography

We verified and quantified the presence of cardenolides in our milkweed bugs and seeds using high performance liquid chromatography (HPLC). Bugs were allowed to feed on *A. syriaca* seeds until the L4 larval stage, after which they were switched to sunflower seeds. After 5 days on sunflower seeds the bugs were frozen at −80 **°**C. To extract the cardenolides the bugs and seeds were first freeze dried and then ground up using a mortar and pistol. The homogenized tissue was then suspended in 2 ml of 100% methanol (with an internal standard of 20 μg digitoxin added to all samples) and heated in a water bath at 55 **°**C or 20 min, followed by 20 min in an ultrasound tissue homogenizer. The methanol was then evaporated in a speedvac for 2 h. Samples were resuspended in 200 μl 100% methanol and then filtered using a 30 mm, 0.45 μm KX Nylon Syringe Filter (Kinesis Scientific Experts, St. Neots, Cambridgeshire, UK).

An Agilent HP1100 HPLC system (Agilent, Waldbronn, Germany) equipped to a C18 column (NUCLEODUR®, Macherey-Nagel) and a diode-array detector was used for separation. We used an injection volume of 15 μl. Samples were analyzed using a gradient elution starting from 16% acetonitrile: 84% H_2_O for 2 minutes and running until 70% acetonitrile: 30% H_2_O for 25 min. The final phase (30 min) and post run (10 min) were run at 95% acetonitrile: 5% H_2_O. The flow rate was held constant at 0.7 ml/min throughout the run. This set up allowed absorbance spectra between 200 and 400 nm to be detected. Peaks having a symmetrical absorbance maximum between 216 and 222 nm were considered to be cardenolides. The majority of cardenolides in plants and insects have not been isolated and described and thus their identity is not known. Retention time on a HPLC column is used as the standard identification technique for these cardenolides, such as that shown here in Fig. 3.

## Results

### Tissue-specific expression pattern

**Fig. 2:**
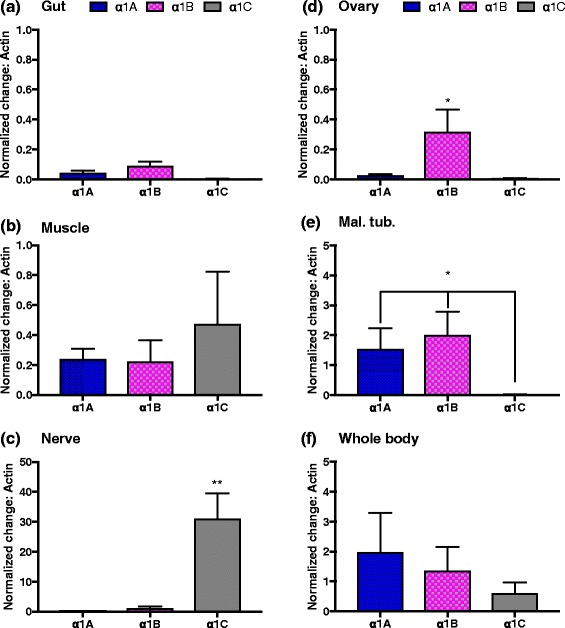
Normalized relative tissue-specific expression of the 3 Na,K-ATPase α-subunit gene copies, relative to the expression of the reference gene Actin in: (**a**) gut, (**b**) muscle, (**c**) nerve, (**d**) ovary, (**e**) Malphigian tubules, and (**f**) whole body. Error bars represent standard errors of mean values for three biological replicates. Note the varying scales on the y-axes

Quantitative RT-PCR revealed differential tissue-specific expression patterns for the 3 Na,K-ATPase α-subunit gene copies (Fig. 2). The α1A was predominantly expressed in the Malpighian tubules, the α1B in the Malpighian tubules and nervous tissue, and the α1C in the nervous and muscle tissue. Particularly striking was that expression of α1C in nervous tissue was over 100 times that of the other copies. There was however, at least some expression of all copies across all tissues.

### *Cardenolide sequestration in O. fasciatus*

**Fig. 3:**
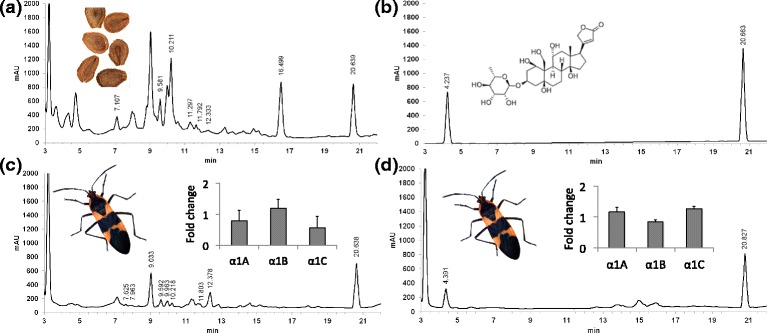
HPLC chromatograms for (**a**) seeds from the milkweed *Asclepias syriaca,* (**b**) a standard sample of the cardenolide ouabain, (**c**) the milkweed bug, *O.fasciatus* after feeding upon *A. syriaca* seeds, and (**d**) *O. fasciatus* after injection with ouabain. Numbered peaks are those identified as cardenolides. The peak at 20 min is that of an internal digitoxin standard of 1.5 μg. (c inset) Change in expression of α1A, α1B and α1C in *O. fasciatus* fed a diet devoid of cardenolides (sunflower seeds) versus milkweed seeds, and (d inset) change in expression when injected with ouabain compared to with a saline solution. Cardenolides that elute at the beginning of the chromatogram are highly polar, whereas those that elute near the end of the chromatogram are highly non-polar. Normalized fold change is relative to the non-cardenolide treatment in both cases. Error bars represent standard errors of mean values for three biological replicates

In order to confirm the presence and determine the number and type of cardenolides sequestered by our milkweed bugs we generated HPLC (high-performance liquid chromatography) profiles of adult bugs and that of their cardenolide containing food source, seeds of the milkweed *Asclepias syriaca* (Fig. 3a,c). We used retention time on the column to identify individual cardenolides, as is standard for such undescribed compounds. We found that though milkweed seeds contain various cardenolides, with a wide range in polarity (Fig. 3a), the milkweed bugs preferentially sequester those with intermediate polarity: those eluting between 7 and 13 min (Fig. 3c). The *A. syriaca* seeds have a strong additional peak at 16.5 min, which is completely absent from *O. fasciatus*, indicating that this cardenolide is either metabolized or excreted. In addition, we took elution profiles of bugs injected with the cardenolide ouabain (Fig. 3b,d) for comparison. Here there was no alteration of the ouabain.

### Expression of the gene copies under cardenolide challenge

After establishing that the milkweed bugs did indeed uptake and sequester cardenolides we then tested for possible up and down regulation of the α1A, α1B and α1C copies after the cardenolide challenge. We did this by measuring the expression levels of the three gene copies via relative RT-qPCR in either control bugs, not challenged with cardenolides, or in bugs fed a diet of milkweed seeds (*A. syriaca*; Fig. 3c inset). Additionally, we recorded the expression changes in bugs injected with either a mock buffer solution or the cardenolide ouabain (Fig. 3d inset). We found no significant evidence for altered gene expression between our treatments, indicating that differential gene regulation does not take place at the mRNA level (Fig. 3c and d insets).

### Phenotypes of knockdowns: Survival and cardenolide challenge assays

**Fig. 4:**
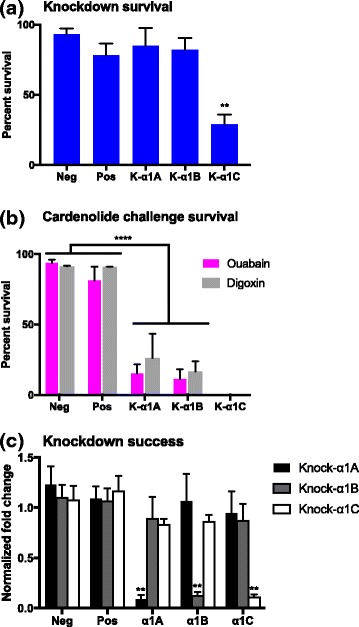
**a** survivorship assay for α1A, α1B and α1C knockdown bugs 5 days post administration of dsRNA. **b** Survivorship assay for bugs post injection with the cardenolides ouabain and digoxin. Neg = negative control, not injected; Pos = positive control, injected with eGFP dsRNA. Error bars represent standard errors of mean values. **c** Efficacy of the sequence-verified and copy-specific RNAi knockdowns for the gene copies α1A, α1B and α1C. Normalized fold change is relative to the eGFP-injected control in each case. Error bars represent standard errors of mean values

We set up five treatment groups: a negative control not injected with anything, a positive control injected with dsRNA for the eGFP gene, and the copy-specific knockdowns for α1A, α1B and α1C. Bugs with successful knockdowns for the α1A and α1B gene copies appeared normal and had a survival rate equal to that of the positive and negative controls (full model: F_4,4_ = 14.2, *P* = 0.012; Tukey HSD comparisons of α1A & α1B to the controls non-significant, with *P* > 0.7 in all cases; Fig. 4a). The α1C knockdowns, on the other hand, displayed difficulties in motor function and had a significantly reduced survival rate compared to the other treatments (full model: F_4,4_ = 14.2, P = 0.012; Tukey HSD comparisons of α1C to Neg: P = 0.01, Pos: *P* = 0.03, α1A: *P* = 0.02, α1B: P = 0.02;). In a second experiment knockdown and control bugs from each RNAi treatment (excluding α1C as knockdowns themselves induced high mortality) were injected with the cardenolides ouabain and digoxin. Both ouabain and digoxin caused significant mortality in the knockdowns as compared to the controls (full model: F_3,8_ = 50.8, *P* < 0.0001; Fig. 4b), though there was no difference in the effects of ouabain and digoxin (interaction factor: F_3,8_ = 0.26, *P* = 0.852). Specifically, the α1A and α1B knockdowns were no longer able to tolerate cardenolides (Tukey HSD comparisons: Neg vs. α1A: *P* = 0.0001, Neg vs. α1B: *P* = 0.001, Pos vs. α1A: *P* = 0.0002, Pos vs. α1B: P = 0.0001).

We verified the success of the RNAi knockdowns via quantitative RT-qPCR, by comparing expression in eGFP injected positive controls with those in the knockdown bugs. A reduction in expression to 10% of the level in the positive controls was considered as a successful knockdown. We also verified that the knockdowns were gene-copy specific by measuring the expression of all gene-copies (Fig. 4c). Fluctuations in expression levels were within a two-fold change margin and thus considered non-significant. RNA levels, however do not always predict protein levels. It depends on the stability of the protein in question how long after mRNA knock-down one will see a decrease in protein level. Here, we see a phenotypic change following mRNA knock-down, which is a confirmation that the knock-down has worked.

## Discussion

Subfunctionalization of gene duplicates is thought to be an important process in the release of genes from pleiotropic effects and thus in facilitating adaptation to novel environmental challenges [20]. Here we show that in the large milkweed bug, *Oncopeltus fasciatus*, multiple gene copies of the Na,K-ATPase α1-subunit (α1A, α1B and α1C), a crucial transmembrane protein, have indeed undergone such subfunctionalization. This result builds on previous work where, using site-directed mutagenesis, it was shown that substitutions present in α1A, α1B and α1C induce different sensitivities to cardenolides [14]. We go on to show that the α1C, which is also the most evolutionarily conserved copy, is highly expressed in nervous tissue, where the pumping function is most important to quickly restore the resting potential of the nerves. In addition, RNAi knockdowns of the α1C copy result in motor dysfunction and death. The other two gene copies, α1A and α1B, are more diverged at the molecular level, and are expressed in the Malpighian tubules, the insect organ responsible for the elimination of toxins. Previous work in *Drosophila* has shown that in these organs the Na,K-ATPase is protected from artificially administered cardenolides by efflux carriers [21, 22]. In *O. fasciatus*, the high concentrations of cardenolides present in its hemolymph have likely made stronger measures of protection necessary and lead to the evolution of highly resistant gene copies.

Our finding that RNAi knockdowns for either of these copies make the bugs less able to tolerate the cardenolides ouabain and digoxin, suggests that the α1A and α1B copies have specialized towards handling these dietary toxins. Importantly, as both the α1A and α1B knock-downs resulted in high mortality, this implies that not only do the two copies serve different functions, but that both are essential to cardenolide tolerance in *O. fasciatus*. They may, for example have subfunctionalized within the Malpighian tubules, where complex processes of excretion and re-absorption are known to occur [23]. Alternatively, α1A and α1B may have different roles in the process of sequestration, for which *O. fasciatus* has evolved a specialized anatomy [24].

Not only do many animals feed on and tolerate cardenolide containing plants, but a large number also sequester the toxins within their own bodies [10]. In *O. fasciatus*, the cardenolides are concentrated and stored within a dorsolateral space along the wall of the cuticle [24, 25]. In fact, members of the Lygaeinae family, such as *O. fasciatus* are among the most effective at concentrating and storing these cardenolides [10, 26]. The sequestration ability of these bugs may be related to the incredible resistance of the α1A, α1B and α1C gene copies, which are also among the most resistant pumps tested to date [26, 27]. Indeed, recent research on milkweed butterflies supports that it is sequestration, and not resistance per se which has been selected for across evolutionary history [28, 29].

The Malpighian tubules are an important part of the excretory system in insects and represent a potential site of metabolism and loss of cardenolides in *O. fasciatus*. Thus, the presence of α1A and α1B in the Malpighian tubules may be related to their elimination after feeding. However, the Malpighian tubules of *O. fasciatus* have not only a distal segment, where cardenolides are indeed secreted into the lumen, but also a proximal segment, where cardenolides are then reabsorbed [23]. Indeed, the Na,K-ATPase has been shown to be involved in fluid transport across the walls of the Malpighian tubules [30], and transporters involved in toxin handling have also been localized to the Malpighian tubules [21, 31, 32]. Thus, it seems likely that cardenolides are shuffled to the Malpighian tubules for excretion and reabsorption, where the highly resistant α1A and α1B can continue to pump in the presence of such high cardenolide concentrations.

Plants produce not just one, but a battery of cardenolides with which their herbivores must cope and thus different mechanisms may be important for the sequestration of diverse cardenolides [10]. Here we show that the bugs preferentially sequester certain cardenolides of the spectrum present in *A. syriaca* and also modify others. Unfortunately, we still have a limited understanding on the physiological effects of different cardenolides. Only a few cardenolides are readily available for use in laboratory experiments: these are ouabain, digoxin and digitoxin. One property, known to be important in cardenolide handling is polarity. Indeed plants contain cardenolides with a wide range of polarities, yet non-polar forms are more prevalent [33]. Here we show that indeed the seeds of *A. syriaca* contain cardenolides with a wide polarity range, whereas the bugs themselves contain less polar forms.

In bugs feeding on milkweed seeds there are cardenolides that elute across a wide range of polarities. However, in the bugs themselves, peaks with a high polarity are totally absent, and instead there are additional peaks of lower polarity. Though this is not definitive evidence of which cardenolides have been converted specifically, it is highly supportive of conversion to and preferential sequestration of lower polarity cardenolides.

The polarity of cardenolides is a large determinant of their toxicity. This is because non-polar cardenolides, such as digoxin and digitoxin, can move with great ease across cell membranes via the transcellular pathway, such as those in the gut [22]. As a result active mechanisms are required to keep these cardenolides out of the body, such as the conversion of non-polar cardenolides into more polar metabolites [24], though how this is accomplished and the pathways involved remains unknown. The use of active efflux transporters for the removal of non-polar cardenolides is another possibility [34]. In contrast protection against polar compounds may be provided by the insect blood-brain barrier, which acts as a barrier via septate and tight junctions [35]. Active transporters located in this barrier, such as ABC (ATP-binding cassette) transporters [36–38] may provide extra protection. However, we still have no experimental evidence for such protection and the additional presence of α1B in nervous tissue found here, suggests that some cardenolides do cross this barrier.

## Conclusions

We show here the use of gene duplication and subsequent subspecialization in facilitating adaptation to an environmental challenge, namely cardenolide ingestion and sequestration. By taking advantage of an insect system where the specific mutations and their effects on cardenolide resistance have been characterized in vitro, we have been able to demonstrate the function of these mutations within duplicated gene copies. These results further our understanding on the function and maintenance of duplicated genes, showing how pleiotropic effects (here cardenolide resistance versus pumping function) can be removed via gene duplication, and how this can facilitate the evolution of increased toxin resistance.

## Additional files

Additional file 1: Sequence alignment. (DOC 62 kb)

Additional file 2: Table S1. Reference gene RT-qPCR primers. E = amplification efficiency, size = size of amplified product. 5’ – 3’ orientation. **Table S2.** RNAi primer pairs used to knockdown the α1A, α1B and α1C gene copies, as well as the eGFP primers used to create the dsRNA for the positive control. 5’ – 3’ orientation. *UniprotKB number. (DOCX 12 kb)

Additional file 3: Figure S1. Gene expression stability and ranking of the seven reference genes as calculated by NormFinder. (a) RNAi treatments, (b) different tissue types, (c) different cardiac glycoside treatments. A lower average expression stability M value indicates more stable expression. (PDF 35 kb)

Additional file 4: Raw Data files. (ZIP 255 kb)
