# Supplementary material for: The function and evolutionary significance of a triplicated Na,K-ATPase gene in a toxin-specialized insect
Source: BMC Evol Biol. 2017 Dec 15;17:256. doi: 10.1186/s12862-017-1097-6 (PMC5732401; doi:10.1186/s12862-017-1097-6)
Supplement: Supplementary file 1 — Sequence alignment. (DOC 62 kb) [file 12862_2017_1097_MOESM1_ESM.doc]

C1 M1

*O. fasciatus* α1A 31 ELDID**Y**HKI**TL**EEL**YR**RF**E**T**N**P**ET**GL**S**H**D**KAKE**I**L**A**RDGPNALTPP**V**TTSEWVKFCKQLF
*O. fasciatus* α1B 31 ELDID**Y**HKI**SL**EEL**FQ**RF**G**T**N**P**ET**GL**T**H**A**KAKE**L**L**E**RDGPNALTPP**K**TTPEWVKFCKQLF
*O. fasciatus* α1C 31 ELDID**H**HKI**SV**EEL**YQ**RF**S**T**H**P**DS**GL**T**H**A**KAKE**N**L**E**RDGPNALTPP**K**TTPEWVKFCKQLF
*D. melanogaster* 31 ELDID**F**HKI**SP**EEL**YQ**RF**Q**T**H**P**EN**GL**S**H**A**KAKE**N**L**E**RDGPNALTPP**K**QTPEWVKFCKNLF
*S. scrofa* 31 KVSMD**D**HKL**SL**DEL**HR**KY**G**T**D**L**SR**GL**T**P**A**RAAE**I**L**A**RDGPNALTPP**P**TTPEWVKFCRQLF

 E1 * * * ** M2 C2
*O. fasciatus* α1A 91 GGFALLLWVGA**I**LCF**V**AY**F**I**TST**T**V**EE**AS**D**NHM**YLGLVLA**G**VVIITG**V**FSYYQE**N**KSSRI
*O. fasciatus* α1B 91 GGFALLLWVGA**A**LCF**I**AY**F**I**TSN**T**E**EE**SS**D**DHM**YLGLVLA**G**VVIITG**I**FSYYQE**N**KSSRI
*O. fasciatus* α1C 91 GGFALLLWVGA**I**LCF**I**AY**S**I**QAT**T**V**EE**PS**D**DHL**YLGIVLA**T**VVIITG**I**FSYYQE**S**KSSRI
*D. melanogaster* 91 GGFAMLLWIGA**I**LCF**V**AY**S**I**QAS**T**S**EE**PA**D**DNL**YLGIVLS**A**VVIVTG**I**FSYYQE**S**KSSKI
*S. scrofa* 91 GGFSMLLWIGA**I**LCF**L**AY**G**I**QAA**T**E**EE**PQ**N**DNL**YLGVVLS**A**VVIITG**C**FSYYQE**A**KSSKI


*O. fasciatus* α1A 151 MESFKNMVPQFA**CV**VRQ**S**EK**I**TIRAE**AL**VLGDVVEVKFGDRIPADIRIIE**S**RGFKVDNSS
*O. fasciatus* α1B 151 MESFKNMVPQFA**IA**VRQ**G**EK**V**TIRAE**EI**VLGDVVEVKFGDRIPADIRIIE**A**RGFKVDNSS
*O. fasciatus* α1C 151 MESFKNMVPQFA**TV**VRQ**G**EK**L**TIRAE**DI**VLGDVVEVKFGDRIPADIRIIE**A**RGFKVDNSS
*D. melanogaster* 151 MESFKNMVPQFA**TV**IRG**G**EK**L**TLRAE**DL**VLGDVVEVKFGDRIPADIRIIE**A**RNFKVDNSS
*S. scrofa* 151 MESFKNMVPQQA**LV**IRN**G**EK**M**SINAE**EV**VVGDLVEVKGGDRIPADLRIIS**A**NGCKVDNSS


*O. fasciatus* α1A 211 LTGESEPQSRG**I**E**M**T**HD**NPLETKNLAFFSTNAVEGTAKG**I**VISCGD**H**TVMGRIAGLASGL
*O. fasciatus* α1B 211 LTGESEPQSRG**I**E**M**T**ND**NPLETKNLAFFSTNAVEGTAKG**V**VISCGD**R**TVMGRIAGLASGL
*O. fasciatus* α1C 211 LTGESEPQSRG**V**E**N**T**HE**NPLETKNLAFFSTNAVEGTAKG**V**VISCGD**N**TVMGRIAGLASGL
*D. melanogaster* 211 LTGESEPQSRG**A**E**F**T**HE**NPLETKNLAFFSTNAVEGTAKG**V**VISCGD**H**TVMGRIAGLASGL
*S. scrofa* 211 LTGESEPQTRS**P**D**F**T**NE**NPLETRNIAFFSTNCVEGTARG**I**VVYTGD**R**TVMGRIATLASGL
 *
 M3 E2 M4
*O. fasciatus* α1A 271 DTG**S**TPIA**R**EI**E**HFIHIITGVA**I**FLG**VS**FF**I**IAF**AM**GY**F**WLDAV**V**FLIGIIVANVPEGLL
*O. fasciatus* α1B 271 DTG**E**TPIA**K**EI**H**HFIHIITGVA**V**FLG**IS**FF**S**IAF**AL**GY**F**WLDAV**V**FLIGIIVANVPEGLL
*O. fasciatus* α1C 271 DTG**E**TPIA**K**EI**H**HFIHIITGVA**I**FLG**IT**FF**I**IAF**LL**GY**Y**WLDAV**I**FLIGIIVANVPEGLL
*D. melanogaster* 271 DTG**E**TPIA**K**EI**H**HFIHLITGVA**V**FLG**VT**FF**V**IAF**IL**GY**H**WLDAV**I**FLIGIIVANVPEGLL
*S. scrofa* 271 EGG**Q**TPIA**A**EI**E**HFIHIITGVA**V**FLG**VS**FF**I**LSL**IL**EY**T**WLEAV**I**FLIGIIVANVPEGLL

 C3
*O. fasciatus* α1A 331 ATVTVCLTLTAKRMAAKNCLVKNLEAVETLGSTSTICSDKTGTLTQNRMTVAHMWFDNQI
*O. fasciatus* α1B 331 ATVTVCLTLTAKRMAAKNCLVKNLEAVETLGSTSTICSDKTGTLTQNRMTVAHMWFDNQI
*O. fasciatus* α1C 331 ATVTVCLTLTAKRMAAKNCLVKNLEAVETLGSTSTICSDKTGTLTQNRMTVAHMWFDNQI
*D. melanogaster* 331 ATVTVCLTLTAKRMASKNCLVKNLEAVETLGSTSTICSDKTGTLTQNRMTVAHMWFDNQI
*S. scrofa* 331 ATVTVCLTLTAKRMARKNCLVKNLEAVETLGSTSTICSDKTGTLTQNRMTVAHMWSDNQI


*O. fasciatus* α1A 391 IEADTTEDQSGVQYD**K**TSPGFKAL**S**RIATLCNRAEFK**P**GQ**E**G**V**PIL**RK**EVNGDASE**S**AL**V**
*O. fasciatus* α1B 391 IEADTTEDQSGVQYD**R**TSPGFKAL**S**RIATLCNRAEFK**P**GQ**D**G**I**PIL**KR**EVNGDASE**A**AL**L**
*O. fasciatus* α1C 391 IEADTTEDQSGVQYD**R**TSPGFKAL**A**RIATLCNRAEFK**G**GQ**E**G**V**PIL**KK**EVNGDASE**A**AL**L**
*D. melanogaster* 391 IEADTTEDQSGVQYD**R**TSPGFKAL**S**RIATLCNRAEFK**G**GQ**D**G**V**PIL**KK**EVSGDASE**A**AL**L**
*S. scrofa* 391 HEADTTENQSGVSFD**K**TSATWLAL**S**RIAGLCNRAVFQ**A**NQ**E**N**L**PIL**KR**AVAGDASE**S**AL**L**


*O. fasciatus* α1A 451 KCMELALGD**IM**SIR**K**RN**K**KVCEIPFNSTNKYQVSIHETEDPND**S**R**Y**LMVMKGAPERIL**E**R
*O. fasciatus* α1B 451 KCMELALGD**IV**SIR**N**RN**K**KVCEIPFNSTNKYQVSIHETEDPND**S**R**Y**LMVMKGAPERIL**D**R
*O. fasciatus* α1C 451 KCMELALGD**VL**SIR**R**RN**R**KVCEIPFNSTNKYQVSIHETEDPND**P**R**H**LMVMKGAPERIL**D**R
*D. melanogaster* 451 KCMELALGD**VM**NIR**K**RN**K**KIAEVPFNSTNKYQVSIHETEDTND**P**R**Y**LLVMKGAPERIL**E**R
*S. scrofa* 451 KCIELAVGX**VK**EMR**E**RY**T**KIVEIPFNSTNKYQLSIHKNPNTAE**P**R**H**LLVMKGAPERIL**D**R


*O. fasciatus* α1A 511 CSTIFIGG**E**EKLLDEE**LRDA**FN**D**AYLELGGLGERVLGFCD**FM**LPPDKFP**V**GF**N**F**NSEE**PN
*O. fasciatus* α1B 511 CSTIFIGG**K**EKLLDEE**MKEA**FN**N**AYLELGGLGERVLGFCD**LL**LPSDKFP**L**GF**D**F**DSED**PN
*O. fasciatus* α1C 511 CSTIFIGG**K**EKLLDEE**MKEA**FN**N**AYLELGGLGERVLGFCD**LM**LPSDKFP**L**GF**K**F**DCDD**PN
*D. melanogaster* 511 CSTIFING**K**EKVLDEE**MKEA**FN**N**AYMELGGLGERVLGFCD**FM**LPSDKYP**N**GF**K**F**NTDD**IN
*S. scrofa* 511 CSSILIHG**K**EQPLDEE**LKDA**FQ**N**AYLELGGLGERVLGFCH**LF**LPDEQFP**E**GF**Q**F**DTDD**VN

*O. fasciatus* α1A 571 FPL**T**G**M**RFVGL**I**SMIDPPRAAVPDAVAKCRSAGIKVIMVTGDHPITAKAIAKSVGIISEG
*O. fasciatus* α1B 571 FPL**T**G**M**RFVGL**I**SMIDPPRAAVPDAVAKCRSAGIKVIMVTGDHPITAKAIAKSVGIISEG
*O. fasciatus* α1C 571 FPL**S**G**L**RFVGL**M**SMIDPPRAAVPDAVAKCRSAGIKVIMVTGDHPITAKAIAKSVGIISEG
*D. melanogaster* 571 FPI**D**N**L**RFVGL**M**SMIDPPRAAVPDAVAKCRSAGIKVIMVTGDHPITAKAIAKSVGIISEG
*S. scrofa* 571 FPL**D**N**L**CFVGL**I**SMIDPPRAAVPDAVGKCRSAGIKVIMVTGDHPITAKAIAKGVGIISEG

*O. fasciatus* α1A 631 NETVEDIA**H**RLNIP**I**SEVNPR**N**AKAAVVHG**TE**L**K**D**IT**PEQLDEILRYHTEIVFARTSPQQ
*O. fasciatus* α1B 631 NETVEDIA**Q**RLNIP**I**SEVNPR**E**AKAAVVHG**SE**L**R**D**TS**PEQLDEILRYHTEIVFARTSPQQ
*O. fasciatus* α1C 631 NETVEDIA**Q**RLNIP**V**SEVNPR**E**AKAAVVHG**TD**L**R**D**TS**PEQLDEILRYHTEIVFARTSPQQ
*D. melanogaster* 631 NETVEDIA**Q**RLNIP**V**SEVNPR**E**AKAAVVHG**AE**L**R**D**VS**SDQLDEILRYHTEIVFARTSPQQ
*S. scrofa* 631 NETVEDIA**A**RLNIP**V**SQVNPR**D**AKACVVHG**SD**L**K**D**MT**SEQLDDILKYHTEIVFARTSPQQ


*O. fasciatus* α1A 691 KLIIVEGCQRMGAIVAVTGDGVNDSPALKKADIG**I**AMGISGSDVSKQAADMILLDDNFAS
*O. fasciatus* α1B 691 KLIIVEGCQRMGAIVAVTGDGVNDSPALKKADIG**V**AMGISGSDVSKQAADMILLDDNFAS
*O. fasciatus* α1C 691 KLIIVEGCQRMGAIVAVTGDGVNDSPALKKADIG**V**AMGISGSDVSKQAADMILLDDNFAS
*D. melanogaster* 691 KLIIVEGCQRMGAIVAVTGDGVNDSPALKKADIG**V**AMGIAGSDVSKQAADMILLDDNFAS
*S. scrofa* 691 KLIIVEGCQRQGAIVAVTGDGVNDSPASKKADIG**V**AMGIAGSDVSKQAADMILLDDNFAS
 *
 M5 *E3 M6
*O. fasciatus* α1A 751 IVTGVEEGRLIFDNLKKSIAYTL**S**SNIPEISPFLA**NVV**L**N**IPLPLG**A**VTILCIDLGTDM**V**
*O. fasciatus* α1B 751 IVTGVEEGRLIFDNLKKSIAYTL**T**SNIPEISPFLA**NVV**L**D**IPLPLG**T**VTILCIDLGTDM**V**
*O. fasciatus* α1C 751 IVTGVEEGRLIFDNLKKSIAYTL**T**SNIPEISPFLA**FMI**L**D**IPLPLG**S**VTILCIDLGTDM**M**
*D. melanogaster* 751 IVTGVEEGRLIFDNLKKSIAYTL**T**SNIPEISPFLA**FIL**C**D**IPLPLG**T**VTILCIDLGTDM**V**
*S. scrofa* 751 IVTGVEEGRLIFDNLKKSIAYTL**T**SNIPEITPFLI**FII**A**N**IPLPLG**T**VTILCIDLGTDM**V**

 C4 M7 E4
*O. fasciatus* α1A 811 PAISLAYE**EA**ESDIMKRQPR**N**P**FS**DKLVN**E**RLIS**LS**YGQIG**V**IQAA**G**GFFVYFVIMAENG
*O. fasciatus* α1B 811 PAISLAYE**EA**ESDIMKRQPR**N**P**FT**DKLVN**E**RLIS**LA**YGQIG**M**IQAA**A**GFFVYFVIMAENG
*O. fasciatus* α1C 811 PAISLAYE**AP**ESDIMKRQPR**D**P**YR**DKLVN**K**RLIS**MA**YGQIG**M**IQAA**A**GFFVYFVIMAENG
*D. melanogaster* 811 PAISLAYE**HA**EADIMKRPPR**D**P**FN**DKLVN**S**RLIS**MA**YGQIG**M**IQAA**A**GFFVYFVIMAENG
*S. scrofa* 811 PAISLAYE**QA**ESDIMKRQPR**N**P**KT**DKLVN**E**QLIS**MA**YGQIG**M**IQAL**G**GFFTYFVILAENG

 M8
*O. fasciatus* α1A 871 FLP**EI**LFGLRK**E**WDSKAVNDL**Q**DSYGQEWTY**KE**RKALE**F**TCHTAFF**IT**IV**I**VQWADLII**S**
*O. fasciatus* α1B 871 FLP**MK**LFGLRK**E**WDSKAVNDL**Q**DSYGQEWTY**RE**RKALE**Y**TCHTAFF**VS**IV**I**VQWADLII**S**
*O. fasciatus* α1C 871 FLP**MK**LFGLRK**S**WDSKAVNDL**L**DSYGQEWTY**QD**RKALE**Y**TCHTAFF**VS**IV**V**VQWADLII**C**
*D. melanogaster* 871 FLP**KK**LFGIRK**M**WDSKAVNDL**T**DSYGQEWTY**RD**RKTLE**Y**TCHTAFF**IS**IV**V**VQWADLII**C**
*S. scrofa* 871 FLP**IH**LLGLRV**N**WDDRWINDV**E**DSYGQQWTY**EQ**RKIVE**F**TCHTPFF**VT**IV**V**VQWADLVI**C**

 C5 M9 E5 * M10
*O. fasciatus* α1A 931 KTRRNSI**I**HQGMRNW**A**LNFGLVFET**V**LAA**F**LSY**C**PGMDKGL**Q**MYPLKFVWWLP**G**LPFM**IV**
*O. fasciatus* α1B 931 KTRRNSI**F**HQGMRNW**A**LNFGLVFET**A**LAA**F**LSY**C**PGMDKGL**R**MYPLKFVWWLP**A**LPFM**VV**
*O. fasciatus* α1C 931 KTRRNSI**F**HQGMRNW**P**LNFGLVFET**A**LAA**L**LSY**T**PGMDKGL**R**MYPLKFVWWLP**A**LPFM**IT**
*D. melanogaster* 931 KTRRNSI**F**QQGMRNW**A**LNFGLVFET**V**LAA**F**LSY**C**PGMEKGL**R**MYPLKLVWWFP**A**IPFA**LA**
*S. scrofa* 931 KTRRNSV**F**QQGMKNK**I**LIFGLFEET**A**LAA**F**LSY**C**PGMGVAL**R**MYPLKPTWWFC**A**FPYS**LL**

 C6
*O. fasciatus* α1A 991 IFIYDEVR**KY**YLRR**Y**PGGWIE**R**ETYY
*O. fasciatus* α1B 991 IFIYDEVR**RF**YLRR**N**PGGWLE**R**ETYY
*O. fasciatus* α1C 991 IFIYDEVR**RF**YLRR**N**PGGWLE**Q**ETYY
*D. melanogaster* 991 IFIYDETR**RF**YLRR**N**PGGWLE**Q**ETYY
*S. scrofa* 991 IFVYDEVR**KL**IIRR**R**PGGWVE**K**ETYY
