# Supplementary material for: The function and evolutionary significance of a triplicated Na,K-ATPase gene in a toxin-specialized insect
Source: BMC Evol Biol. 2017 Dec 15;17:256. doi: 10.1186/s12862-017-1097-6 (PMC5732401; doi:10.1186/s12862-017-1097-6)
Supplement: Supplementary file 2 — Reference gene RT-qPCR primers. E = amplification efficiency, size = size of amplified product. 5’ – 3’ orientation. Table S2. RNAi primer pairs used to knockdown the α1A, α1B and α1C gene copies, as well as the eGFP primers used to create the dsRNA for the positive control. 5’ – 3’ orientation. *UniprotKB number. (DOCX 12 kb) [file 12862_2017_1097_MOESM2_ESM.docx]

Additional file 2

Table S1 Reference gene RT-qPCR primers. E = amplification efficiency, size = size of amplified product. 5’ – 3’ orientation

| Gene | Forward primer | Revers primer | E (%) | Size (bp) |
| --- | --- | --- | --- | --- |
| Actin | TGCACCAAGGGCTGTCTTCC | TTGGGCCTCATCACCGACATAG | 90.0 | 103 |
| β-tub | TGCCTGGTT TTGCAC CACTGAC | GGGTCACAAGCG GCCATCAT | 92.3 | 115 |
| Ef1A | CCACCAAGGGCTGCTTCTGA | GCAATATGGGCTGTGTGGCAAT | 96.2 | 110 |
| COI | GCACCTGATATAGCATTTCCACGA | CAGTTCATCCTGTTCCAGCTCC | 100.0 | 118 |
| GAPDH | GAATGCCATGCCAGTTAGTTTGC | GTTGTGGCGTGATGGGAGAG | 98.1 | 115 |
| RPS3 | TTGACTCGGGAACTTGCTGAAGA | TCACCTAAGACACTTTGGGTACGA | 96.6 | 110 |
| 18S | AGGGACAGGCGGCTCTTAG | CAGGGGAGCACACGCTGATT | 99.8 | 112 |

Table S2 RNAi primer pairs used to knockdown the α1A, α1B and α1C gene copies, as well as the eGFP primers used to create the dsRNA for the positive control. 5’ – 3’ orientation. *UniprotKB number

| Gene | Accession # | Fwd primer | Rvs primer | Product size (bp) |
| --- | --- | --- | --- | --- |
| α1A | JQ771520 | AGGAAAGGAAAGCATTAGAGTT | CACTGATAAATTGACATCAG | 540 |
| α1B | JQ771519 | GAGAAAGGAAGGCATTAGAGTA | GTACTTTTAGAGGGGCC | 542 |
| α1C | JQ771518 | AAGATAGAAAAGCGCTTGAGTAT | AACAGCTAATTGATAAACAG | 533 |
| eGFP | C8CHS1* | CGTAAACGGCAAGTTCAG | TGAAGTTCACCTTGATGCCG | 433 |
