# Supplementary figures and images for: The function and evolutionary significance of a triplicated Na,K-ATPase gene in a toxin-specialized insect
Source: BMC Evol Biol. 2017 Dec 15;17:256. doi: 10.1186/s12862-017-1097-6 (PMC5732401; doi:10.1186/s12862-017-1097-6)

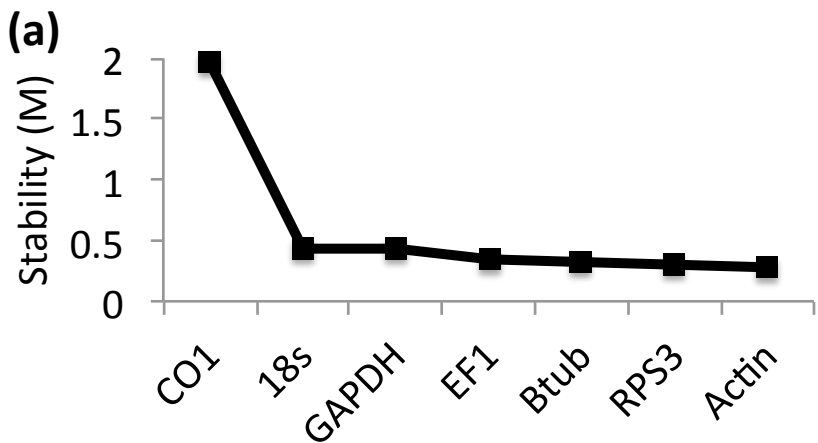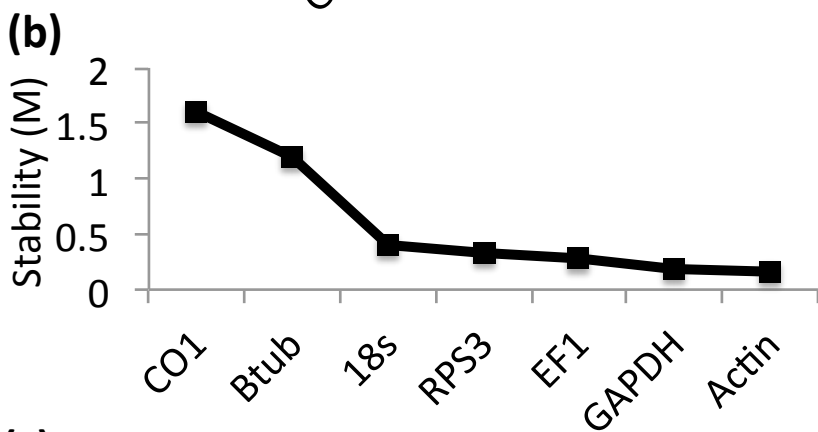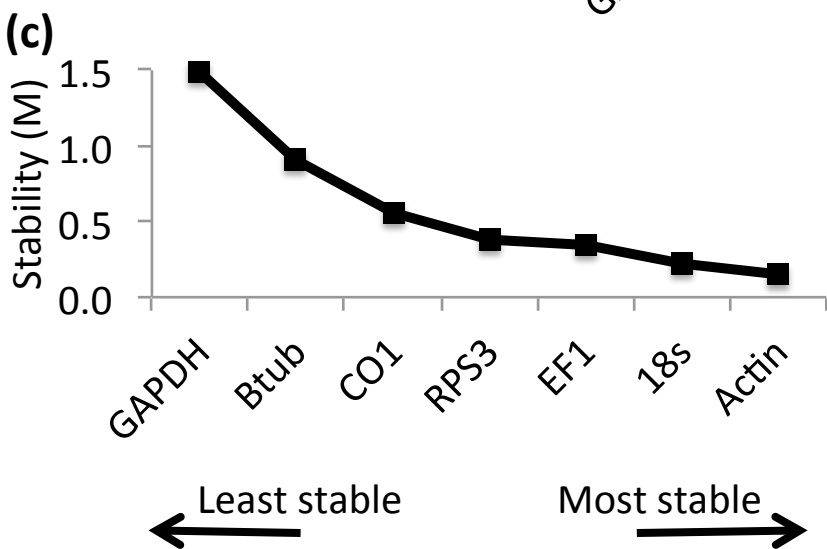

Supplement: Supplementary file 3 — Gene expression stability and ranking of the seven reference genes as calculated by NormFinder. (a) RNAi treatments, (b) different tissue types, (c) different cardiac glycoside treatments. A lower average expression stability M value indicates more stable expression. (PDF 35 kb) [file 12862_2017_1097_MOESM3_ESM.pdf]
